# Randomized control trial on the efficacy of *Limosilactobacillus reuteri* ATCC PTA 4659 in reducing inflammatory markers in acute uncomplicated diverticulitis

**DOI:** 10.1097/MEG.0000000000002342

**Published:** 2022-01-17

**Authors:** Veronica Ojetti, Angela Saviano, Mattia Brigida, Carmine Petruzziello, Martina Caronna, Gunawardena Gayani, Francesco Franceschi

**Affiliations:** a Emergency Department – Fondazione Policlinico Universitario A. Gemelli, IRCCS, Roma; b Emergency Department – Università Cattolica del Sacro Cuore, Roma; c Ospedale San Carlo di Nancy GVM care and Research, Rome

**Keywords:** acute uncomplicated diverticulitis, inflammatory biomarkers, *Limosilactobacillus reuteri* 4659

## Abstract

Recent guidelines suggest treating acute uncomplicated diverticulitis (AUD) without antibiotics. We tested the efficacy of *Limosilactobacillus reuteri* ATCC PTA 4659 in AUD. Primary outcome was the reduction of abdominal pain and inflammatory markers [C-reactive protein (C-RP) and calprotectin]. Secondary outcome was the reduction of hours of hospitalization.

**Patients and methods:**

A double-blind, randomized controlled trial was conducted in 119 patients with AUD. The probiotic group (61 patients) was treated with fluids, bowel rest and *L. reuteri*/b.i.d. for 10 days. The placebo group (58 patients) was treated with the same therapy and placebo/b.i.d. for 10 days. All patients completed a daily visual analogue scale (VAS) for abdominal pain.

**Results:**

Both groups showed a mean VAS score of 7 at enrolment and a reduction of 4 points after 3 days. C-RP value, after 72 h, decreased by 58.8% in the probiotic group and by only 40% in the placebo group (*P* < 0.05). Calprotectin levels, after 72 h, decreased by 17% in the probiotic group and by only 10.6% in the control group (*P* < 0.05). In the probiotic group, the hospitalization was done for 75.5 h compared to 83.5 in the placebo group.

**Conclusions:**

The supplementation with *L. reuteri* 4659 together with bowel rest and fluids significantly reduced both blood and faecal inflammatory markers compared to the placebo group.

## Introduction

The presence of diverticula along the large bowel is a very common disease, especially in Western countries and the elderly population [1–4]. The most frequent complication, occurring in up to one-fifth of patients, is the occurrence of an acute attack of diverticulitis [5–7]. The clinical spectrum of the disease varies from a mild isolated ‘attack’ of peridiverticular inflammation [acute uncomplicated diverticulitis (AUD)] to very severe forms of diverticular inflammation acute complicated diverticulitis with perforation and abscesses with high morbidity and mortality [1].

Patients with acute diverticulitis typically present with a sudden onset of abdominal pain, usually in the lower left part requiring access to the Emergency Department (ED), usually have fever and increased levels of inflammatory markers [white blood cells and C-reactive protein (C-RP)]. The gold standard for the diagnosis is the computed tomography (CT) fundamental for a correct differential diagnosis [colon cancer and inflammatory bowel disease (IBD)] and to assess the degree of severity and to guide clinical management.

Recent guidelines suggest that AUD should be treated conservatively (without antibiotic therapy), with just rest, liquid diet and observation. The vast majority of patients with AUD respond to therapy within a few days [3].

Older patients, or patients with significant comorbidities, must be hospitalized and receive intravenous antibiotic treatment.

At the base of diverticular disease, there seems to be the alteration of the intestinal bacterial flora which plays a fundamental role, resulting in an increased production of intestinal gas, favouring a state of chronic microinflammation and predisposition to visceral hypersensitivity.

Gut microbiota differs qualitatively and quantitatively in various parts of the intestine. The microflora has trophic and protective functions, contributes to the regulation of intestinal permeability, is involved in the processes of immunomodulation and influences the motility of the gastrointestinal tract [8].

Among the 500–1000 species of our intestinal microbiota, there are useful bacteria that do not induce an inflammatory response and do not activate the signalling pathways that lead to the secretion of tumour necrosis factor-α (TNF-α); but there are also pathogenic bacteria able to bind to Toll-like receptors (TLRs) on cell membranes which, through the MyD88 protein, dephosphorylate the nuclear factor κB (NFκB) inhibitor (IκB) allowing the latter to migrate into the nucleus and lead to an overexpression of TNF-α and interleukin-8 (IL-8) generating proinflammatory effects. In this way, a Th17-type inflammatory response is activated which is also able to influence the enteroglia and the signal transmission pathways, and therefore pain, at the submucosa level. Therefore, in the presence of a balanced flora and a healthy environment, inflammatory reactions in the intestine do not occur and therefore, there is a state of well-being and health; if this balance is altered such as in bacterial overgrowth or dysbiosis, an inflammatory state is generated which could be responsible for symptomatic diverticular diseases. Thus, the intestinal microbiota should become a specific target for therapy in these pathological conditions. In the etiopathogenesis of acute diverticulitis, an altered bacterial flora can determine, through an inflammatory state, altered activation of the afferent and efferent fibers with a relative muscular and neuronal dysfunction, which leads to the development of abdominal symptoms [2]. Sopeña and Lanas suggest that the presence of a bacterial flora with a high fermentation and a high production of gas results in the distension of the intestinal lumen with consequent development of symptoms. Antibiotics and probiotics are the main agents able to modify the balance of the intestinal microbiota. Antibiotics should act by reducing the bacterial load and therefore the fermentation processes, the production of gas, to reduce the intraluminal pressure and thus the presence of symptoms [4].

In the context of diverticular disease, probiotics are useful because they counteract the adhesion of harmful bacteria to the intestinal mucosa, modify the metabolic aspects at the mucosal level and reduce the synthesis of inflammatory cytokines [9].

One of the most studied probiotics in the literature and most effective in reducing antibiotic-associated diarrhoea, traveller’s diarrhea and infantile colic is *Limosilactobacillus reuteri* [6].

There are different strains of *L. reuteri* on the market with specific peculiarities of action.

*L. reuteri* ATCC PTA 4659 (*L. reuteri* 4659) showed a potent anti-inflammatory action by inhibiting experimental colitis in IL-10-deficient transgenic mice, while also reducing the levels of proinflammatory cytokines such as TNF-α [6]. These studies have, therefore, shown the therapeutic potential of the strain of *L. reuteri* 4659 in IBDs, in children with Crohn’s disease and in the treatment of ulcerative proctitis.

Recently, our group showed in a randomized controlled trial (RCT) that in patients with AUD, the supplementation with *L. reuteri* 4659 to the standard antibiotic therapy significantly reduced abdominal pain and inflammatory markers compared with the placebo group. It also resulted in a shorter period of hospitalization and thus has economic benefits.

Another study confirmed that the supplementation with a mix of probiotics with an anti-inflammatory effect, during antibiotic treatment for AUD, is able to quickly reduce abdominal pain, inflammation and duration of hospitalization [10].

Based on the new guidelines on the clinical management of AUD, we decided to evaluate the impact of the supplementation with probiotics in reducing inflammatory biomarkers in the ED.

## Study objective

The objective of the present study was to evaluate the efficacy of the supplementation with *L. reuteri* 4659 together with bowel rest and fluids in the treatment of AUD in EDs.

The primary endpoint was the modification of inflammation markers in blood sample and stool specimen, and the reduction of clinical symptoms in the group receiving the probiotic, compared with the group of patients treated with placebo.

The secondary endpoint was the reduction in hours of hospitalization in the group receiving the probiotic, compared with the placebo group.

### Patients and methods

A double-blind, placebo-controlled, randomized trial was conducted (October 2019–December 2020) on 109 consecutive adult patients (44 M/65 F; mean age 65.1 ± 20.0 years) with a diagnosis of AUD (Hinchey = 0) admitted to the ED of Fondazione Policlinico Universitario A. Gemelli IRCCS, Rome, Italy.

The inclusion criteria were age greater than 18 and less than 80 years, fever less than 38 °C, C-RP less than 180 mg/L, no reported allergies to contrast agents or antibiotics, informed consent and diagnosis of AUD confirmed by abdomen CT scan (Hinchey = 0). The exclusion criteria were age less than 18 or greater than 80 years, fever greater than 38 °C, C-RP greater than 180 mg/L, prior colonic surgery, pregnancy or breastfeeding, concomitant or recent (7–10 days) participation in another clinical trial, concomitant or recent (7–15 days) intake of probiotics or antibiotics, major concurrent diseases (hepatological, renal and tumour), IBD (Crohn’s disease, ulcerative colitis) or other organic gastrointestinal disease, allergies to contrast agents or antibiotics and mental illness or inability to adhere to protocols. Withdrawal criteria from the study: at the request of the patient, evidence of side effects related to the drugs administered, appearance of septic state, increase of C-RP greater than 180 mg/L, appearance of fever greater than 38.5 °C and development of complications.

Patients were evaluated in ED or brief observation unit by a physician at the enrolment into the study, every day during hospitalization and at the end of therapy. At enrolment, a medical history review (including drugs taken), physical examination, laboratory tests (blood cell count, hepatic and renal function, electrolytes and C-RP), stool sample (calprotectin level) and abdominal CT scan were performed.

All patients presented with AUD (Hinchey classification grade 0) [5]. All patients were given a visual analogue scale (VAS) ranging from 0 to 10, where 0 is asymptomatic and 10 is the worst pain they could have, to complete during the 10 days of the study.

Patients maintained a diary to record any ‘adverse experience’ (causing discomfort and interrupting the subject’s usual activity) during the treatment period and to record each time they did not consume the prescribed doses. The diary was analysed by physicians.

**Fig. 1.**
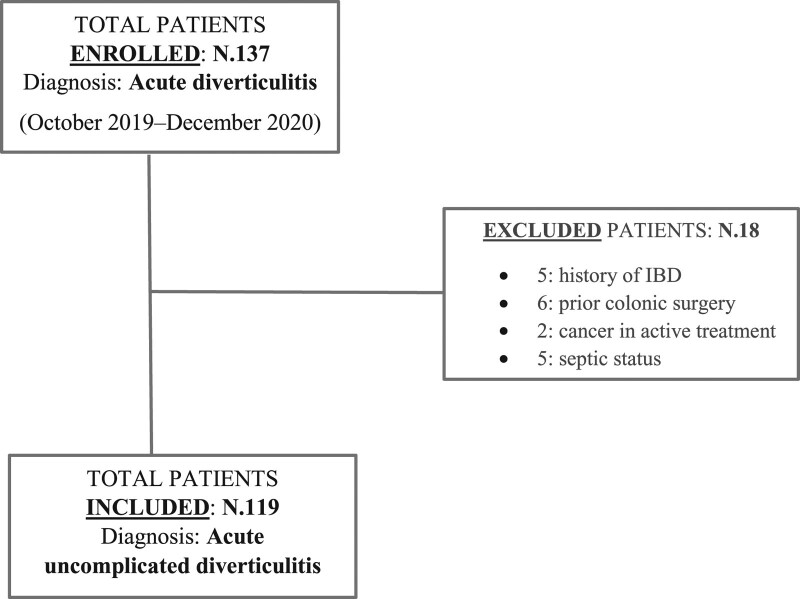
Study flowchart. IBD, inflammatory bowel disease.

During the enrolment period, 137 patients obtained a confirmed diagnosis of acute diverticulitis in ED; 18 did not meet the inclusion criteria, in particular, five patients with a history of IBD, six patients with a history of prior colonic surgery, two patients with a history of cancer in active treatment and five patients with a septic status [5] (Fig. 1).

The 119 patients who met the inclusion criteria were randomly assigned into two groups, according to an automatically generated randomization list in a 1:1 ratio, using statistical software.

About 18 patients (15%) have had a previous episode of AUD. The most common comorbidities found were cardiovascular diseases (hypertension, atrial fibrillation, heart failure, etc.), type 2 diabetes, dementia, reflux disease and liver/gallbladder diseases (in few cases).

The probiotic group (*n* = 61 patients 36 F) received intravein fluids (Isolyte 2000 in 24 h), bowel rest for 48 h and a supplementation with the probiotic *L. reuteri* 4659 twice a day for 10 days.

The placebo group (*n* = 58 patients 34 F) received the same standard therapy as the probiotic group and a matching placebo for the same periods.

After 72 h of treatment, C-RP and calprotectin levels were measured.

Calprotectin level was determined in a few minutes with CalFast XT (Eurospital SPA, Trieste, Italy) that applies immunochromatographic technology to a solid support (Card) on which the stool sample was placed; the colored bands that appeared in the Card were detected and quantified by a special reader (CalFast Reader). The quantification of calprotectin was performed by referring to a standard curve residing inside the reader which is automatically inserted by reading the specific bar code on the package with an optical pen (CalFast Bar Code Reader). The normal value is considered less than 50.

Patients were informed by an investigator (blinded) that such a supplement could help in improving the inflammation associated with diverticulitis. Boxes containing placebo had the same shape dimensions, and trademark indication and contained the same number of capsules as *L. reuteri* boxes, and they were provided by the same probiotic producer.

The supplement of *L. reuteri* 4659 was administered in a dose of 5 × 10^8^ colony-forming units, in capsules in morning and night. During the study period, patients were instructed to store the product according to the recommended temperature. In particular, the capsules have a long shelf life at room temperature (25 °C) but because *L. reuteri* is a living organism, the study product was kept refrigerated (2–8 °C) to minimize any differences in viability of the bacteria during the course of the trial.

Finally, protocol adherence was verified through a capsule count of the boxes returned by subjects on the day after finishing the therapy and by directly asking the subjects about completion of the therapy.

All patients gave written informed consent. The study was approved by the independent Ethics Committee of the Catholic University of Rome (ID 1398) and conducted in accordance with the Declaration of Helsinki. Subjects did not receive any payment for their participation in the study. Intention to treat and per protocol analysis was performed.

### Statistical analysis

The data analysis included a descriptive part of the sample which was carried out by building frequency tables (absolute and percentage) for the categorical variables and by calculating the mean ± SD and the 95% confidence interval for the quantitative variables.

Based on the pilot study ‘The efficacy of Lactibiane Iki (*Bifidobacterium lactis La 304, Lactobacillus salivarius La 302 and Lactobacillus acidophilus La 201*) in reducing abdominal symptoms and inflammatory biomarkers in acute uncomplicated diverticulitis’ in which we observd an average reduction after 5 days of VAS in the control group of 7.5 (SD: 1.1), and in the treated groups of 4.1 (SD: 0.8) and setting as error alpha 0.05 and error beta 0.80, it will be necessary to enrol 43 patients to arm, for a total of 86 patients. Considering a dropout rate of 5%, it was decided to sample 90 subjects (with a ratio treated:controls of 1:1).

The VAS scale is a typical ordinal variable (with a minimum value of 0 corresponding to the absence of pain and a maximum value of 10, corresponding to extreme pain). There will be a total of 10 administrations of the VAS scale. For each administration, and separately for cases and controls, the following measurements will be calculated: minimum, maximum, range, mode and median. These values will be graphically represented to give a visual idea of the pain trend in patients. The comparison will be made with Friedman’s test, a nonparametric version of the repeated measures analysis of variance.

## Results

The two study groups were randomly well matched for age, sex and grade of initial inflammation (mean C-RP value and calprotectin and mean VAS at enrolment) (Table 1).

**Table 1.** Main characteristics of probiotic group and placebo group patients at enrolment

	Probiotic group (*Limosilactobacillus reuteri* 4659; 5 × 10 CFU)	Placebo group (placebo)	*P* value
Sex (M/F)	25 M/36 F	24 M/34 F	ns
Mean age (years)	63.8 ± 11.5	67.02 ± 12.8	ns
VAS at enrolment Range: 0 (asymptomatic)–10 (worst pain)	Score: 7	Score: 7	ns
C-RP (mg/dl) at enrolment	57.0 + 22.5	37.71 + 11.2	*P* < 0.05
Faecal calprotectin level (mg/L) at enrolment	640.0 + 150.20	568.20 + 130.65	ns

CFU, colony-forming unit; C-RP, C-reactive protein; F, female; M, male; VAS, visual analogue scale.

In the randomization, we observed that the C-RP value (mg/dl) at enrolment was statistically significantly higher in the probiotic group compared to the placebo group (57.0 + 22.5 vs. 37.71 + 11.2, respectively, *P* < 0.05).

We analysed data of 61 patients from the probiotic group (25 M and 36 F) mean age 63.8 ± 11.5 years and 58 patients from the placebo group (24 M and 34 F) mean age 67.02 ± 12.8 years (Fig. 1).

Most of our patients showed an AUD of the left side and just eight patients present an extension of the diverticula also in the right colon.

### Abdominal pain assessed on a visual analogue scale

**Fig. 2.**
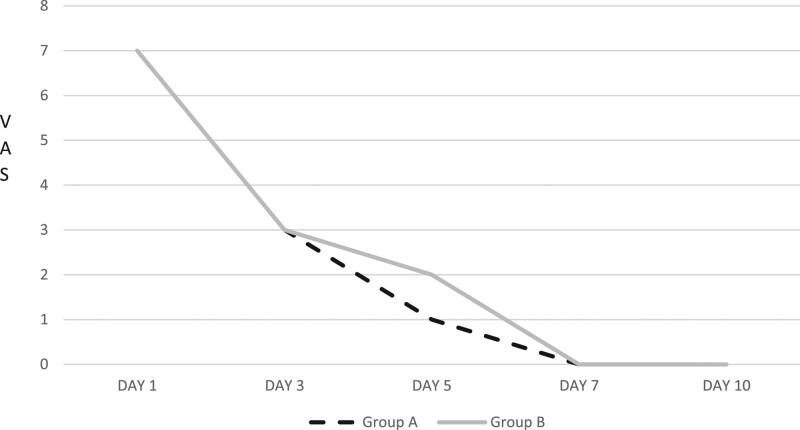
Mean rating of abdominal pain as assessed by the visual analog scale (VAS) completed by subjects in groups A (*Limosilactobacillus reuteri*) and B (placebo) on Days 1, 3, 5, 7 and 10.

On Day 1, the two groups showed the same VAS score, with a mean of 7 points for both groups (Fig. 2).

On Day 3, the probiotic group showed a mean VAS score of 3 points and the control group showed a value of 3.

On Day 5, the mean VAS score was 1 point in the probiotic group compared to 2 points in the control group.

On Day 7, both groups reported a mean VAS of 0 points.

### C-reactive protein level

**Fig. 3.**
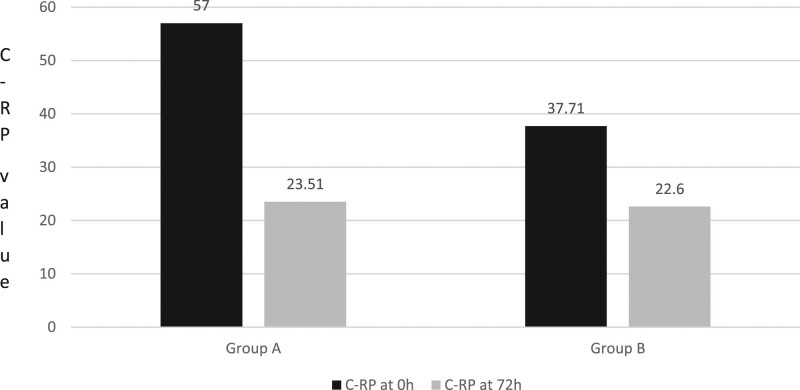
Mean value of C-reactive protein (C-RP, mg/L) determined at enrollment and at 72 h in subjects of groups A (*Limosilactobacillus reuteri*) and B (placebo).

At enrolment, C-RP level has a mean of 57.0 ± 22.5 mg/dl in the probiotic group and 37.71 ± 11.2 mg/dl in the control group (Fig. 3).

At 72 h, the probiotic group showed a mean C-RP value of 23.51 ± 10.05 mg/dl compared to a mean level of 22.6 ± 8.4 mg/dl in the control group (*P* < 0.05). Therefore, the decrease in the inflammatory marker was 58.8% in the probiotic group and only 40% in the control group.

### Calprotectin level

**Fig. 4.**
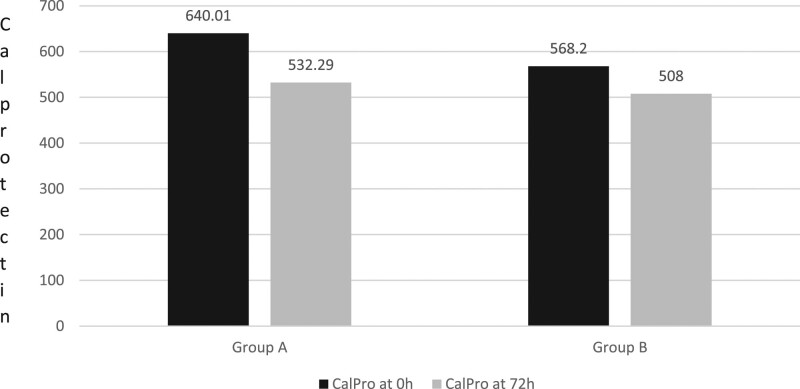
Mean value of calprotectin determined at enrollment and at 72 h in subjects of groups A (*Limosilactobacillus reuteri*) and B (placebo).

We compare the mean change in C-RP, at enrolment and after 72 h from hospital admission (Fig. 4).

At enrolment, the mean calprotectin level of the probiotic group was 640.01 ± 150.20 mg/L; meanwhile, the control group showed a mean of 568.20 ± 130.65 mg/L.

At 72 h, the calprotectin level in the probiotic group decreased to a mean of 532.29 ± 110.64 mg/L and to 508.0 ± 101.9 mg/L in the control group.

The calprotectin levels decreased by 17% in the probiotic group, whereas it decreased by only 10.6% in the control group (*P* < 0.05).

### Duration of hospitalization

Patients of the probiotic group stayed in the hospital for an average of 75.47 ± 15 h, whereas the mean duration of hospitalization of patients in the control group was 83.5 ± 16.5 h.

## Discussion

Our RCT shows that the supplementation with the probiotic *L. reuteri* 4659 for 10 days in patients with AUD is able to quickly reduce both blood and faecal inflammation markers and the duration of hospitalization.

Recently, we conducted another RCT in patients with AUD. They received the supplementation with the same strain of *L. reuteri* (4659) and the standard antibiotic therapy, with a significant reduction of abdominal pain and inflammatory markers compared with the placebo group (antibiotics only). It also resulted in an economic benefit for the hospital and the healthcare system as it reduced the period of hospitalization.

In our trial, we observed that patients treated with *L. reuteri* 4659 had a reduction in C-RP level of around 70% compared to 40% in the placebo group after 72 h (*P* < 0.05). This datum is even more relevant considering that the initial C-RP values were higher in the group treated with probiotics compared to the placebo group.

Many literature studies showed that C-RP is a good diagnostic indicator in acute diverticulitis, helping to discriminate complicated from uncomplicated forms [11]. Complicated forms have significantly higher C-RP levels compared to patients with the uncomplicated disease [12]. Moreover, C-RP is a useful serum marker, not only in the diagnostic phase but also in the prediction of acute diverticulitis’ severity progression [13]. The monitoring of C-RP values can give an idea of the course of the disease [13]. The reduction, during days, in C-RP level may indicate that the disease and the grade of inflammation are improving, with benefits for patients.

Recent pieces of evidence in literature [14] suggest that inflammation is strongly related to changes in gut microbiota composition, known as dysbiosis. In this context, the supplementation with probiotics in patients with acute diverticulitis can help in restoring the colonization of colon and diverticula by anti-inflammatory species as *Lactobacilli* and *Bifidobacteria*, which resulted in alleviation of this disease. Enterobacteriaceae, *Streptococcus* and *Bacteroides* level resulted increased in patients with acute diverticulitis.

This dysbiosis, as discussed previously, might be linked to mucosal inflammation, and a vicious cycle results from a mucosal inflammation driving dysbiosis at the same time [14]. Probiotics may be effective in ‘breaking’ this vicious cycle, modifying the course of this disease.

Moreover, alteration in gut microbiota composition can lead to altered activation of nerve fibres and subsequent neuronal and muscular dysfunction, thus favouring the development of abdominal symptoms. The bacterial translocation from the lumen of the diverticulum to perivisceral area is responsible for the activation of TLRs, with a subsequent inflammatory reaction of the perivisceral tissues.

Despite these aspects, in our study, no differences were observed in the two groups as regards the abdominal pain evaluated troughs VAS score, during the first days of treatment. In fact, we registered a reduction of 4 points of the VAS scale after 72 h from enrolment in both groups.

In our opinion, in case of uncomplicated diverticulitis with mild inflammation without abscesses, perforation or other complications, bowel rest and fluids are able to improve patient’s symptoms as suggested by the recent guidelines. A study by Fric *et al*. [15] showed that the supplementation with *Escherichia coli* strain Nissle 1917, a well-known probiotic with anti-inflammatory effect in patients with AUD, was able to improve gastrointestinal symptoms after 7 days of treatment.

Many factors contribute to the pathogenesis of abdominal symptoms (that include pain, vomiting, bloating, nausea, borborygmus, etc.) as inflammation of the gut mucosa, alteration in gut motility and neuronal dysfunction, besides the central sensitization [16]. Probiotics, as shown by different studies [16], can modulate all of these aspects, but this process needs time. Tursi *et al*. [17] conducted a prospective randomized multicenter study on 90 patients with AUD. These patients were randomized into three groups: mesalamine, probiotic (*L. casei*) or a combination of probiotic and mesalamine. The group treated with the combination of mesalamine and *L. casei* for 10 days/month for 12 months showed a total absence of symptoms compared to the other two groups.

In our study, patients treated with *L. reuteri* revealed a lower value of VAS score compared to placebo groups on the fifth day of treatment (1 vs. 3). So, the action of probiotics on abdominal complaints needs time in consequence that many different factors are involved in the pathogenesis of abdominal pain.

About faecal calprotectin, we observed that patients with AUD had a high level at the moment of diagnosis (around 10 times the normal value), and just a mild reduction after 72 h. We expected this result because the reduction of local inflammation in the gut needs more time. The administration of probiotics can speed this process but, in a time, longer than 3 days.

This study is the first that evaluates the level of faecal calprotectin (an antimicrobial protein belonging to the group of Ca^2+^-binding proteins of the S100 family) in patients affected by AUD at diagnosis in ED. Faecal calprotectin is present in neutrophilic granulocytes where it represents 5% of the total proteins and 60% of the portion of cytoplasmic proteins; to a lesser extent, it is found in activated macrophages and monocytes. The increase in faecal calprotectin is due to the faecal excretion of neutrophils and macrophages migrated from the bloodstream into the intestinal lumen through the inflamed mucosa.

It has been proven that the faecal calprotectin level correlates with the histological and endoscopic picture of intestinal inflammation in patients with IBD but little data are available about patients with colonic diverticular disease.

Tursi *et al*. [18] performed a case–control study on faecal calprotectin levels in different phases of colonic diverticular disease; they observed normal value in asymptomatic diverticulosis, healthy controls and irritable bowel syndrome patients (*P* = ns). Meanwhile, a higher faecal calprotectin value was observed in AUD and in symptomatic uncomplicated diverticular disease (SUDD), and this correlated with inflammatory infiltrate (*P* < 0.0005). Faecal calprotectin decreased to normal values both in AUD (*P* < 0.0005) and SUDD (*P* < 0.005) after the treatment.

The ability of *L. reuteri* 4659 to decrease plasma C-RP and faecal calprotectin levels contributes to allowing a more rapid healing and a faster discharge from the hospital. In fact, we found that patients of the probiotic group stayed in the hospital for an average of 75.47 + 15 h, whereas the mean duration of hospitalization in patients of the control group was 83.5 ± 16.5 h, with economic benefits for the healthcare centre.

Limitations of the study were the absence of multivariate analysis and the absence of an evaluation of the gut microbiota and the prevalence of different microbial species in the acute phase of the disease. Previous literature studies have suggested a reduction of the anti-inflammatory taxa (as *Clostridium cluster IV*, *Bacteroides* and *Lactobacilli*). At the same time, the overgrowth of *Akkermansia*, *Bifidobacteria* and Enterobacteriaceae has been reported. It could be interesting to evaluate if the supplementation with probiotics is able to switch this ‘unbalanced’ gut microbiota composition in AUD. More studies are needed to explore this issue.

### Conclusion

In conclusion, based on the results of our study, we suggest starting as soon as possible, in patients with AUD, a supplementation with *L. reuteri* 4659 together with fluids and bowel rest to improve the course of the disease, gut inflammation and to prevent complications as suggested by the most updated guidelines. Moreover, other studies are needed to complete and investigate these results in a larger sample of patients.

## Acknowledgements

### Conflicts of interest

There are no conflicts of interest.
